# Evaluation of Characteristics and Outcomes of COVID-19 Between First Wave and Second Wave: Study From a Tertiary Cancer Care Centre, Delhi, India

**DOI:** 10.7759/cureus.35386

**Published:** 2023-02-23

**Authors:** Atika Dogra, Vidya Krishna, Anuj Parkash, Anurag Mehta, Tarun Varma

**Affiliations:** 1 Department of Research, Rajiv Gandhi Cancer Institute and Research Centre, Delhi, IND; 2 Department of Research and Development, Rajiv Gandhi Cancer Institute and Research Centre, Delhi, IND; 3 Department of Biochemistry, Rajiv Gandhi Cancer Institute and Research Centre, Delhi, IND; 4 Department of Laboratory and Transfusion Services and Department of Research, Rajiv Gandhi Cancer Institute and Research Centre, Delhi, IND; 5 Department of Internal Medicine, Rajiv Gandhi Cancer Institute and Research Centre, Delhi, IND

**Keywords:** second wave, first wave, critical care, covid-19, case fatality ratio

## Abstract

Background and objectives

The second wave of coronavirus disease-19 (COVID-19) had several severe consequences in the form of rising cases, deaths, and overwhelming health infrastructure in India. However, the similarities and differences between the characteristics of the first and second waves have yet to be explained. The objectives of the study were to compare the incidence, clinical management, and mortality rates between two waves.

Methods

The COVID-19 data collated from Rajiv Gandhi Cancer Institute and Research Centre, Delhi between the first wave (1 April 2020 to 27 February 2021) and second wave (1 March 2021 to 30 June 2021) were evaluated in terms of incidence, the clinical course of the disease, and mortality rates.

Results

The number of subjects hospitalized in the first and second waves was 289 and 564, respectively. Compared to the first wave, the proportion of patients with severe disease was higher (9.7% vs. 37.8%). Several parameters such as age group, grade of disease, the reason for hospitalization, values of peripheral oxygen saturation, type of respiratory support, response to therapy, vital status, and others show statistically significant differences between the two waves (P<0.001). The mortality rate in the second wave was significantly higher (20.2% vs. 2.4%, P<0.001) than in the first wave.

Interpretation and conclusions

The clinical course and outcomes of COVID-19 significantly differ between the first and second waves. There is a higher incidence of hospitalized patients (66.1% vs. 33.9%) with drastically increased case fatality rate in the second wave. Disease severity in the first wave is four times lower than in the second wave. The second wave was quite devastating, which led to the shortage of critical care facilities and the loss of a significant number of lives.

## Introduction

Novel coronavirus disease-19 (COVID-19), caused by severe acute respiratory syndrome coronavirus 2 (SARS-CoV-2), has been affecting many lives since its emergence as a pandemic. India has witnessed three major waves of the COVID-19 pandemic and has been the second worst coronavirus-hit country after the US [1].

The second wave of COVID-19 had severe consequences as rising cases, reduced supplies of essential treatments, increased deaths in the young population, and overwhelmed health infrastructure across the country [2]. However, similarities and differences between the characteristics of the two waves in the Indian population have not been investigated much. This study aims to compare the clinical course and outcomes of COVID-19 between the first wave and the second wave. The objectives are to compare the incidences, grades of disease severity, and mortality rates between two waves.

## Materials and methods

The study was granted a waiver (letter number RGCIRC/IRB-BHR/133/2021) by our Institutional Review Board. The participants’ data were anonymized, so the requirement for informed consent was waived.

Study design

This was a retrospective and observational study. The duration of the first and second waves was defined to be from 1 April 2020 to 27 February 2021 and from 1 March 2021 to 30 June 2021, respectively [3]. In our center, the first case of the first wave was admitted on 8 June 2020 and the last case of the second wave was admitted on 8 June 2021.

Case selection

The inclusion criterion in this study was to include all COVID positive cases admitted to our Institution for COVID treatment and care, except for cancer patients. A confirmed COVID-19 case was defined as a positive SARS-CoV-2 reverse transcription polymerase chain reaction (RT-PCR) on a nasal and throat swab or a CT scan of the chest consistent with COVID-19 (a CO-RADS score of ≥ 4 along with the clinically diagnosed viral pneumonia). A total number of 886 patients with COVID-19 were admitted to our hospital in the first and second waves combined. Thirty-three patients were excluded because some patients’ electronic medical records were not available and a few had malignancy, hence 853 cases were included in this study.

Data collation

Detailed information regarding the patient’s age, gender, underlying disorders, symptoms, laboratory data, treatment, respiratory support, need for intensive care, duration of illness, secondary infection during the hospital stay, and disease outcome at the time of hospital discharge was gathered from the electronic medical records of subjects. According to the criteria of the Indian Council of Medical Research (ICMR), mild, moderate, and severe diseases were defined by upper respiratory tract symptoms (and/or fever) without dyspnea or hypoxia; respiratory rate >24/min, breathlessness, and/or SpO_2_ 90% to <93% on room air; respiratory rate >30/min, breathlessness and/or SpO_2_ <90% on room air, respectively [4]. Time to diagnosis was calculated as the days between the onset of the first symptom and to date of diagnosis. The time to admission was computed as the duration from the date of diagnosis to hospitalization. Time to death was calculated as the number of days between the dates of symptoms’ onset and death.

Statistical analysis

The statistical analyses of data were done using Statistical Package for the Social Sciences (SPSS) (IBM SPSS Statistics for Windows, Version 23.0, Armonk, NY). The statistical summary was presented in mean±SD (standard deviation) or percent as per the distribution of data. The quantitative variables of two waves were compared using independent samples Student’s ‘t’ test/Mann-Whitney U test. Chi-squared test/Fisher’s exact tests were used to compare the categorical variables. The results with a P value <0.05 were accepted as statistically significant. The log-rank test was applied to compare Kaplan-Meier curves for survival analysis.

## Results

Demographic and clinical characteristics of the entire cohort of patients have been mentioned (Table 1). The most commonly affected age group was those aged between 46 and 60 years. The incidence of disease was higher in males compared to females. Among patients who had co-morbid conditions, the majority (51.8%) had a single underlying condition, followed by two (33.7%), three (11.2%), and four or more (3.3%). The patients predominantly reported flu-like symptoms (96.5%) followed by desaturation (64.6%), anosmia/ageusia (2.7%), loss of appetite (2.2%), and others (2.1%). Breathlessness was the major reason for hospitalization. Respiratory support was provided to 485 (56.9%) cases as per their clinical requirements. The requirement for an intensive care unit was observed in 156 (18.3%) cases; however, all of them couldn’t get critical care as per their need because of the non-availability of beds. Among all hospitalized patients, 121 (14.2%) didn’t respond to any therapy and lost their lives. Of all patients, 23 (3.6%) patients had developed a secondary infection as bacterial and/or fungal infections.

**Table 1:** Demographic and clinical characteristics of cases. SD - Standard deviation, RT-PCR - Real-time-polymerase chain reaction, CBNAAT - cartridge-based nucleic acid amplification test, COVID-19 Ag - Novel coronavirus disease Antigen, HRCT - high resolution computed tomography, mAb - monoclonal antibody, ICU - intensive care unit

Characteristic (N=853)	Frequency (%)
Age (years)	
Mean (SD)	50.9 (16.9)
Median (range)	52 (18-88)
Sex	
Female	351 (41.1)
Male	502 (58.9)
Co-morbidities	
Absent	451 (52.9)
Present	402 (47.1)
Type of investigation	
RT-PCR	712 (83.5)
CBNAAT	79 (9.3)
Covid-19Ag test	44 (5.1)
HRCT	18 (2.1)
Grade of symptoms	
Mild	321 (37.6)
Moderate	291 (34.1)
Severe	241 (28.3)
Wave (853)	
First	289 (33.9)
Second	564 (66.1)
Reason for admission	
Breathlessness	517 (60.6)
Fever and/or cough	149 (17.5)
Digestive symptoms	8 (0.9)
Others	18 (2.1)
Precautionary	161 (18.9)
Type of therapy	
Glucocorticoids	611 (71.6)
Anticoagulants	729 (85.5)
Antiviral drugs	559 (65.5)
Antibiotics	522 (61.2)
mAb therapy	70 (8.2)
Plasma therapy	87 (10.2)
Respiratory support	
Oxygen by mask or cannula	358 (42.0)
Non-invasive ventilation	76 (8.9)
Mechanical ventilation	51 (6.0)
Not given	368 (43.1)
ICU admission	
Not required	697 (81.7)
Given as required	124 (14.5)
Required but not available	32 (3.8)
Response to therapy	
Recovered/Improved	723 (84.7)
Expired	121 (14.2)
Not known	9 (1.1)
Secondary infection	
Absent	830 (97.3)
Bacterial	14 (1.6)
Fungal	3 (0.4)
Both	6 (0.7)
Vital status	
Alive	732 (85.8)
Dead	121 (14.2)

There were 289 (33.9%) and 564 (66.1%) cases in the first and second waves, respectively, as shown in Table 1. The mean age of patients was lower in the first wave as compared to the second wave that occurred during March-June 2021 (46.91 ± 18.71 years vs. 52.95 ± 15.77 years, P<0.001). Also, the most frequently infected age group in the first wave was between 15 and 30 years compared to those aged 46-60 years in the second wave (P<0.001). The comparison of categorical variables between the two waves has been illustrated (Table 2). The difference between the investigation types of disease detection between the two waves was highly significant (P<0.001). High-resolution computed tomography (HRCT) was also used for diagnosis in the cases of the second wave, where the results of RT-PCR were false negative. The grade of disease severity mostly belongs to moderate and severe (77.9%) in the second wave of spring 2021, however; in the first wave, it was chiefly mild. The main reason for hospitalization in the second wave was oxygen desaturation; however, most hospitalizations in the first wave were done as a precautionary measure. Among all the treatments, anticoagulants were given to many patients in both waves. To summarize, all studied parameters showed highly statistically significant differences (P<0.001) except gender, co-morbidities, and secondary infection during the comparison of categorical variables between the two waves (Table 2).

**Table 2:** Comparison of categorical variables between two waves. RT-PCR - real-time-polymerase chain reaction, CBNAAT - cartridge-based nucleic acid amplification test, COVID-19 Ag - Novel coronavirus disease Antigen, HRCT - high resolution computed tomography, mAb - monoclonal antibody, ICU - intensive care unit

Characteristic (N=853)	First wave (n=289)	Second wave (n=564)	Chi-square	P value
Age group (years)				
15-30	80 (27.7)	44 (7.8)	64.801	<0.001
31-45	63 (21.8)	152 (27)		
46-60	62 (21.5)	172 (30.5)		
61-75	70 (24.2)	143 (25.4)		
>75	14 (4.8)	53 (9.4)		
Sex				
Female	127 (43.9)	224 (39.7)	1.411	0.235
Male	162 (56.1)	340 (60.3)		
Co-morbidities				
Absent	161 (55.7)	290 (51.4)	1.412	0.235
Present	128 (44.3)	274 (48.6)		
Investigation type				
RT-PCR	272 (94.1)	440 (78)	47.158	<0.001
CBNAAT	14 (4.8)	30 (5.3)		
Covid-19Ag test	3 (1)	76 (13.5)		
HRCT	0 (0)	15 (2.7)		
Grade of symptoms				
Mild	196 (67.8)	125 (22.2)	176.477	<0.001
Moderate	65 (22.5)	226 (40.1)		
Severe	28 (9.7)	213 (37.8)		
Desaturation				
Absent	173 (59.9)	129 (22.9)	114.319	<0.001
Present	116 (40.1)	435 (77.1)		
Reason for admission				
Breathlessness	88 (30.4)	429 (76.1)	200.627	<0.001
Fever/cough	70 (24.2)	79 (14.0)		
Precautionary	121 (41.9)	40 (7.1)		
Digestive symptoms	2 (0.7)	6 (1.1)		
Others	8 (2.8)	10 (1.8)		
Glucocorticoids				
Given	113 (39.1)	498 (88.3)	227.591	<0.001
Not given	176 (60.9)	66 (11.7)		
Anticoagulants				
Given	196 (67.8)	533 (94.5)	109.512	<0.001
Not given	93 (32.2)	31 (5.5)		
Antiviral drugs				
Given	107 (37)	452 (80.1)	157.281	<0.001
Not given	182 (63)	112 (19.9)		
Antibiotics				
Given	106 (36.7)	416 (73.8)	110.643	<0.001
Not given	183 (63.3)	148 (26.2)		
mAb therapy				
Given	5 (1.7)	65 (11.5)	24.336	<0.001
Not given	284 (98.3)	499 (88.5)		
Plasma therapy				
Given Not given	14 (4.8) 275 (95.2)	73 (12.9) 491 (87.1)	13.685	<0.001
Respiratory support				
Oxygen by mask/cannula	58 (20.1)	300 (53.2)	235.766	<0.001
Non-invasive ventilation	2 (0.7)	74 (13.1)		
Mechanical ventilation	1 (0.3)	50 (8.9)		
Not required	228 (78.9)	140 (24.8)		
ICU admission				
Given as required	29 (10)	95 (16.8)	26.136	<0.001
Required but not available	0 (0)	32 (5.7)		
Not required	260 (90)	437 (77.5)		
Response to therapy				
Recovered	278 (96.2)	445 (78.9)	49.826	<0.001
Expired	7 (2.4)	114 (20.2)		
Not known	4 (1.4)	5 (0.9)		
Secondary infection				
Absent	285 (98.6)	545 (96.6)	2.869	0.90
Present	4 (1.4)	19 (3.4)		
Vital status				
Alive	282 (97.6)	450 (79.8)	49.683	<0.001
Dead	7 (2.4)	114 (20.2)		

The comparison of numerical variables between the two waves has been presented (Table 3). The time to diagnosis and hospitalization in the first wave was lower than in the second wave (P<0.001). The values of all laboratory results were also significantly different between the two waves, excluding Interleukin-6 and serum creatinine. Similarly, the score of HRCT and the percentage of oxygen saturation were also statistically different between the two waves (Table 3).

**Table 3:** Comparison of numerical variables between two waves. SD - standard deviation, HRCT - high resolution computed tomography, SpO_2_ - saturation of peripheral oxygen, SGOT - serum glutamic-oxaloacetic transaminase, SGPT - serum glutamic pyruvic transaminase, ICU - intensive care unit

Characteristic characteristic	First wave		Second wave		P value
	Number	Mean (SD)	Number	Mean (SD)	
Age (years)	289	46.91 (18.17)	563	52.95 (15.77)	<0.001
Time to diagnosis (days)	289	3.79 (2.34)	564	4.76 (3.04)	<0.001
Time to hospitalization (days)	269	1.87 (2.56)	541	3.78 (4.05)	<0.001
C-Reactive protein (mg/dL)	225	29.92 (143.78)	545	78.47 (439.11)	<0.001
D-Dimer (ng/ml)	280	520.63 (2395.83)	554	1107.60 (4697.20)	<0.001
Interleukin-6 (pg/ml)	25	1356.16 (2242.27)	200	739.59 (1717.77)	0.438
HRCT score	79	7.848 (6.40)	302	14.225 (6.12)	<0.001
Minimum SpO_2_ without support (%)	289	93.05 (5.35)	506	86.93 (10.91)	<0.001
Minimum SpO_2_ with support (%)	68	91.41 (7.58)	424	82.17 (17.51)	<0.001
Serum SGOT level	282	56.36 (149.79)	545	74.05 (142.58)	.049
Serum SGPT level	282	55.60 (140.30)	545	67.51 (69.92)	<0.001
Serum Ferritin level (ng/ml)	77	620.73 (1372.48)	218	840.91 (2220.14)	<0.001
Blood Urea	243	34.47 (73.5)	542	59.37 (315.07)	<0.001
Serum Creatinine	244	8.65 (10.11)	543	9.64 (14.61)	0.060
Total bilirubin	282	5.52 (4.07)	536	6.86 (15.32)	0.033
ICU stay (days)	27	9.22 (6.48)	92	10.37 (8.90)	0.876
Hospital stay (days)	289	6.54 (4.52)	564	8.87 (7.68)	<0.001
Time to death (days)	9	19.44 (12.88)	112	18.82 (9.58)	0.855

**Figure 1:**
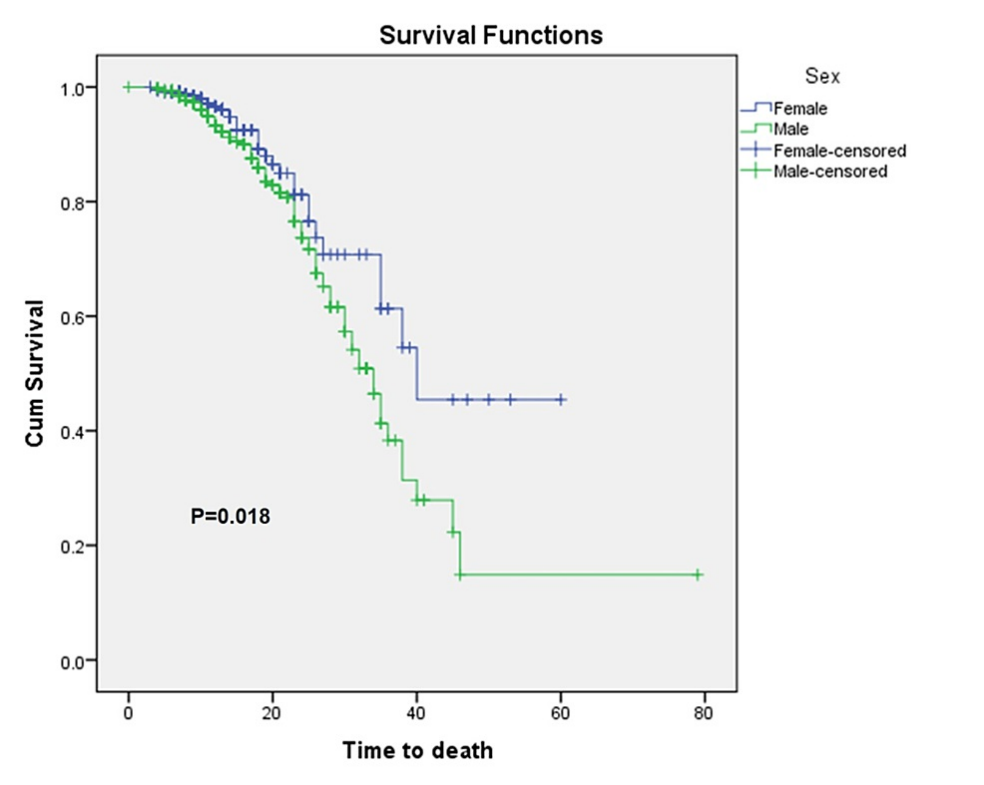
Kaplan-Meier survival curve for time to death corresponding to sex. Censored - Either patient is lost to follow-up or the event does not occur within the study duration, Cum - cumulative

**Figure 2:**
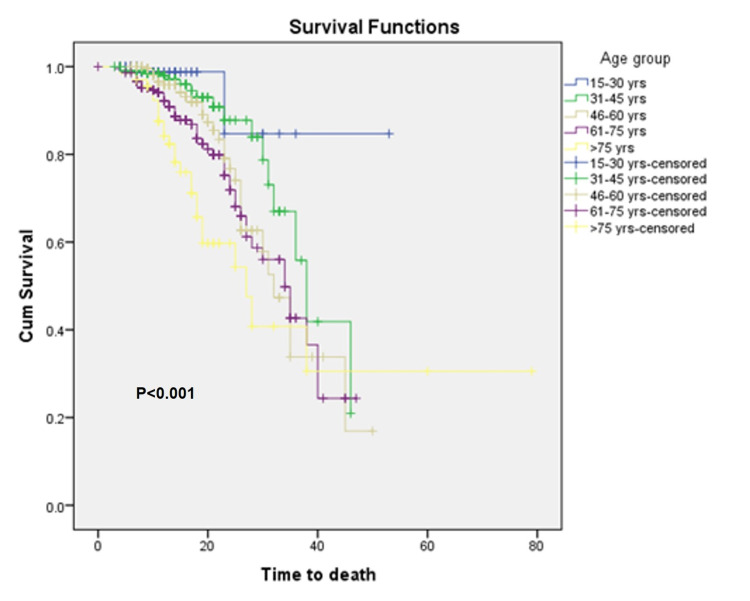
Kaplan-Meier survival curves comparing time to death corresponding to different age groups. Censored - Either patient is lost to follow-up or the event does not occur within the study duration, Cum - cumulative

**Figure 3:**
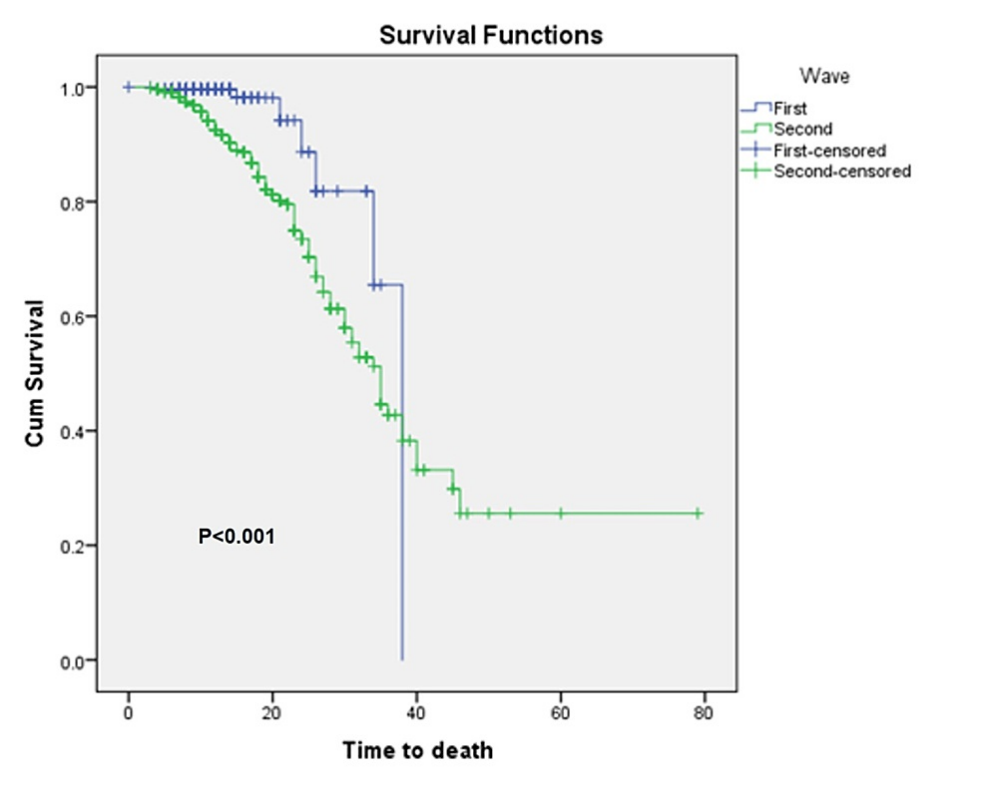
Kaplan-Meier survival curves for comparing time to death between two waves. Censored - Either patient is lost to follow-up or the event does not occur within the study duration, Cum - cumulative

Mortality rates in the first and second waves were 2.4% and 20.2%, respectively, and response to therapy was highly distinct (P<0.001). The case fatality ratio (CFR) was higher in males (82.1%) compared to females (91.2%) in the combined data set of two waves. Kaplan-Meier survival analysis confirmed the time to death was significantly lower (P=0.018) among males compared to their female counterparts (Figure 1). As revealed (Figure 2), the time to death was lowest in the age group of 46-60 years. Furthermore, the age groups had significantly different times of death (P<0.001) among themselves (Figure 3). The difference in time to death between the two waves was also highly significant (P<0.001).

## Discussion

In this study, we have described and compared the demographic, clinical characteristics, and mortality of hospitalized COVID-19-infected patients between the first and second waves of the pandemic. The study reflects differences in the number and characteristics of hospitalized patients between the two waves. The frequency of patients admitted to our center during the first wave was half of that in the second wave. It is primarily related to the high transmissibility of the Delta variant (B.1.617.2) which was first detected in India in December 2020 and became the most commonly reported variant in the country from mid-April 2021 [5]. Shiehzadegan et al. reported that more than 26% of the Indian population was infected with the delta variant in just three months [6].

The number of male patients was higher compared to females [7]. The higher CFR and shorter time to death among males provide evidence that men with COVID-19 are more susceptible to worse outcomes and death, which corroborates with the literature [8]. In addition, the lower time to death among males indicates that men died earlier than women. The median age observed in our study is comparable to the findings reported by Kong TK [9], but there is variation across demographic regions [10].

The lower time to diagnosis in the first wave (3.8 vs. 4.8 days) may be because people were more cautious about detection in the first wave due to the fear of novel disease compared to the second, though the delta-driven second wave during March-June 2021 was highly transmissible and deadlier than the first. The longer time for hospitalization in the second wave may be because people had learned how to deal with the disease from the first wave and waited until the emergence of unmanageable symptoms at home. The number of secondary infections in both the first and second waves combined was lower than that reported by Ripa et al. [11].

Cases falling in the age group of 46-60 years were hospitalized the most. The trend of increasing CFR was observed with an increase in age and was the highest (35.8%) in the group of cases aged >75 years. This finding coincides with the existing literature [12]. Interestingly, there was not a single case of hospitalization of children in our center during both waves. It shows that children were, in one way or another, protected from the worst of this disease [13]. In the second wave, HRCT was also used to detect the disease in patients who missed diagnosis via RT-PCR, and the disease testing by the Covid-19Ag test was also done more in the second wave of spring 2021. The reason behind this may be that there was a high load for RT-PCR testing and a longer waiting time for report generation as a result of the massive surge in cases in the second wave and suspected cases were opting for other alternatives too for testing. The significant difference between types of investigation may also be attributable to this.

There was a significant difference in the causes of hospitalization between the two waves. More than two-thirds of cases in the second wave had experienced dyspnea. This may be related to the pathogenesis of variant B.1.617.2 by which mutated spike protein increases its binding affinity to angiotensin-converting enzyme 2 (ACE2) receptors. This leads to higher replication efficiency in human lung epithelial cells and causes pneumonia [5]. The higher values of laboratory tests such as C-reactive protein, D-dimer, serum ferritin, and others (Table 3) in the second wave that occurred during March-June 2021 are connected with the severity and poor outcomes of the disease. These have been recognized as independent risk factors for severity in COVID-19 cases. The higher mean 25-point CT score in the second wave (14.23 vs. 7.85), lower SpO_2_ levels with (82.17% vs. 91.41%), and without oxygen support (86.93% vs. 93.05%) reinforce the second wave's aggressiveness. This is consistent with the findings reported in the literature [14].

The difference in the prescribed treatments between the two waves is in concordance with that reported by Carbonell et al. [15]. Increased use of corticosteroids, antiviral drugs, and antibiotics in the second wave corresponds to having a high number of moderate and severe cases in this subgroup. This is supported by another finding that while only one patient (0.3%) required mechanical ventilation in the first wave, 50 (8.9%) cases needed it in the second wave of spring 2021. Interestingly, the length of ICU stay between the two waves did not differ significantly, despite having a significant variation in hospital stays. In the first wave, all patients had received the required treatment and clinical care, however; 32 patients couldn’t get admission to ICU in the second wave. It reflects how healthcare facilities were overwhelmed with patients and the existing resources were depleted beyond capacity. In our study, the CFR in the second wave was significantly higher than in the first wave, which proves that the former was quite frightening and devastating. The major cause of higher CFR in the second wave was the higher proportion of cases with moderate and severe disease caused by the COVID-19 variant, Delta. Compared to previously reported wild-type variants, this particular strain had been more contagious and associated with significantly higher mortality [16]. Even essential and critical care tools were not reachable many times and the people couldn’t help themselves, gasping for oxygen or medicine and rushing for hospital beds [17]. Though various places like open fields, religious places, and others were converted into makeshift wards and centers for accessing oxygen [17], basic amenities were not available to several needy patients. Many young individuals have died in the second wave, and it has affected almost everyone since a close family member or acquaintance died during the pandemic. Many people couldn’t get to see their loved ones for the last time and the grief load had increased owing to a lack of a grieving and mourning process.

In the current study, we could not examine the association between vaccination and disease outcome as the information regarding vaccination was available in the records of very few patients. In addition, assessing the Omicron variant-induced third wave was not achievable in this study because the infections were mild and the number of hospitalizations was very low during this wave.

## Conclusions

The present study results show significant differences in the clinical management and outcomes of COVID-19 between the two waves in India. In the second wave, the incidence of hospitalized patients was much higher, with drastically increased CFR in the second wave. The middle-aged (46-60 years) group was the most infected, but the mortality rate was highest in the elderly (>75 years). The frequency of severe and critical cases was higher in the second wave and the prescribed treatments also significantly differed compared to the first wave. There was a shortage of critical care facilities during the second pandemic wave. Males infected with COVID-19 are more susceptible to worse clinical outcomes and death.

The virus still exists and keeps gathering new mutations, its future waves may have more severe outcomes. Hence, it is necessary to keep up the mask and follow COVID-19-appropriate behavior. Viral evolution will remain a concern for a future resurgence.
